# Care pathways for critically ill children aged 0–5 years arriving at district hospitals in Burkina Faso, Guinea, Mali, and Niger (2022): a cross-sectional study

**DOI:** 10.1186/s12889-025-24835-1

**Published:** 2025-11-13

**Authors:** Emelyne Gres, Sarah Louart, Bertrand Meda, Lucie Peters-Bokol, G. Désiré Kargougou, Gildas Boris Hedible, Abdoul-Guaniyi Sawadogo, Zineb Zair, Jacques Séraphin Kolié, Emmanuel Bonnet, Valéry Ridde, Valériane Leroy, AIRE Research Group, S. Yugbaré Ouédraogo, V. M. Sanon Zombré, M. Sama Cherif, I. S. Diallo, D. F. Kaba, A. A. Diakité, A. Sidibé, H. Abarry Souleymane, F. T. Issagana Dikouma, H. Agbeci, L. Catala, D. L. Dahourou, S. Desmonde, E. Gres, L. Peters Bokol, J. Tavarez, A. Cousien, R. Becquet, V. Briand, V. Journot, S. Lenaud, B. Seri, C. Yao, G. Anago, D. Badiane, M. Kinda, D. Neboua, P. S. Dia, S. Shepherd, N. di Mauro, G. Noël, K. Nyoka, W. Taokreo, O. B. Coulidiati Lompo, M. Vignon, P. Aba, N. Diallo, M. Ngaradoum, S. Léno, A. T. Sow, A. Baldé, A. Soumah, B. Baldé, F. Bah, K. C. Millimouno, M. Haba, M. Bah, M. Soumah, M. Guilavogui, M. N. Sylla, S. Diallo, S. F. Dounfangadouno, T. I. Bah, S. Sani, C. Gnongoue, S. Gaye, J. P. Y. Guilavogui, A. O. Touré, A. S. Savadogo, F. Sangala, M. Traore, T. Konare, A. Coulibaly, A. Keita, D. Diarra, H. Traoré, I. Sangaré, I. Koné, M. Traoré, S. Diarra, V. Opoue, F. K. Keita, M. Dougabka, B. Dembélé, M. S. Doumbia, S. Keita, S. Bouille, S. Calmettes, F. Lamontagne, K. H. Harouna, B. Moutari, I. Issaka, S. O. Assoumane, S. Dioiri, M. Sidi, K. Sani Alio, S. Amina, R. Agbokou, M. G. Hamidou, S. M. Sani, A. Mahamane, A. Abdou, B. Ousmane, I. Kabirou, I. Mahaman, I. Mamoudou, M. Baguido, R. Abdoul, A. Sahabi, F. Seini, Z. Hamani, M. Niome, X. Toviho, I. Sanouna, P. Kouam, R. Abdoulaye-Mamadou, S. Busière, F. Triclin, A. Hema, M. Bayala, L. Tapsoba, J. B. Yaro, S. Sougue, R. Bakyono, A. Soumah, Y. A. Lompo, B. Malgoubri, F. Douamba, G. Sore, L. Wangraoua, S. Yamponi, S. I. Bayala, S. Tiegna, S. Kam, S. Yoda, M. Karantao, D. F. Barry, O. Sanou, N. Nacoulma, N. Semde, I. Ouattara, F. Wango, Z. Gneissien, H. Congo, Y. Diarra, B. Ouattara, A. Maiga, F. Diabate, O. Goita, S. Gana, S. Diallo, S. Sylla, D. Coulibaly, N. Sakho, K. Kadio, J. Yougbaré, D. Zongo, S. Tougouma, A. Dicko, Z. Nanema, I. Balima, A. Ouedraogo, A. Ouattara, S. E. Coulibaly, H. Baldé, L. Barry, E. Duparc Haba, A. Coulibaly, T. Sidibe, Y. Sangare, B. Traore, Y. Diarra, A. E. Dagobi, S. Salifou, B. Gana Moustapha Chétima, I. H. Abdou

**Affiliations:** 1 Department of Molecular Medicine, University of Padua, Padua, Italy; 2 Department of Women’s and Children’s Health, Division of Pediatric Infectious Diseases, University Hospital of Padua, Padua, Italy; 3 CERPOP, UMR 1295, Inserm, University of Toulouse 3, Toulouse, France; 4 Center for Epidemiology and Research in Population Health (CERPOP), Inserm, Université Toulouse, Toulouse, France; 5 UMR 8019 - CLERSE - Centre Lillois d’Études Et de Recherches Sociologiques Et Économiques, Univ. Lille, CNRS, Lille, 59000 France; 6 Solthis, Niamey, Niger; 7 ALIMA, Bamako, Mali; 8 Tdh, Ouagadougou, Burkina Faso; 9 ALIMA, Conakry, Guinea; 10 UMR 215 PRODIG, IRD, CNRS, Université Paris 1 Panthéon-Sorbonne, Paris, France; 11 Université Paris Cité, IRD, Inserm, Ceped, Paris, 75006 France

**Keywords:** Child health, Pathway of care, Primary health care, Referral to hospital, Burkina Faso, Guinea, Mali, Niger

## Abstract

**Background:**

Under-five mortality remains high in West Africa, where sick children are expected to first attend the primary health care before being referred to a hospital if necessary. However, little is known about how families navigate between home and higher levels of care to meet their children’s health needs, despite multiple known barriers (including social, financial, and geographical accessibility). We analysed the care pathways of children aged 0–5 years before they presented to the district hospital with a serious illness and the determinants of these care pathways in four West African countries.

**Methods:**

From May to August 2022, we conducted a cross-sectional study over a one-month data collection in seven district hospitals participating in the AIRE project aimed to introduce pulse oximetry at primary health care level in Burkina Faso, Guinea, Mali, and Niger. All children aged 0–5 years, classified as severe or priority cases by clinicians at referral district hospitals were included after parental consent. Data about care pathways since the onset of their disease were collected from caregivers, and the Levesque framework was used to analyse the accessibility issues.

**Results:**

A total of 861 severely ill children were included, with 33% being neonates: 20.3% in Burkina Faso, 9.2% in Guinea, 9.5% in Mali, and 61% in Niger. In Burkina Faso and Niger, most children followed the recommended care pathway and first visited a primary health centre before arriving at the hospital, with 81.1% and 73.3% of children, respectively. However, they were only 51.2% in Mali and 13.9% in Guinea. Using alternative pathways was common, particularly in Guinea, where 30.4% of children first consulted a pharmacist, and Mali, where 25.6% consulted a traditional medicine practitioner. Overall, primary care was perceived to be more geographically accessible and less expensive, but parents were much less convinced that it could improve their child's health compared to hospital care.

**Conclusion:**

The recommended pathway is largely adhered to, yet parallel pathways require attention, notably in Guinea and Mali. A better understanding of healthcare-seeking behaviours can help remove barriers to care, improving the likelihood that a sick child will receive optimal care.

**Supplementary Information:**

The online version contains supplementary material available at 10.1186/s12889-025-24835-1.

## What is already known on this topic:

- In West Africa, public health services are planned hierarchically: sick children are expected to attend first the first level of primary health care before being referred to upper levels, if necessary…
- The health system in this part of the world generally consists of a combination of public and private care, alongside a traditional system that includes traditional medicine and alternative practitioners.
- Access to appropriate care for children under the age of five is often not guaranteed due to several barriers (transport, cost, quality of care, etc.).

## What this study adds:

- There has been relatively little research exploring how barriers to access to care differ between facilities and influence care pathways.
- This study illustrates the diversity of family care pathways in four West African countries with different health policies and varying barriers to care.

## How this study might affect research, practice, or policy:

- Understanding the behaviour of families when seeking care makes it possible to act on the main obstacles to access to care
- Free-of-charge healthcare policies appear to significantly impact on access to health centres and the use of recommended pathways. It is therefore important to maintain and ensure these policies where they exist, and to extend them where they do not yet exist.
- It is also crucial to improve the quality of primary care, which is more accessible and less expensive, but in which parents have much less confidence in improving their children's care

## Introduction

Under-five mortality remains high in West Africa, despite significant progress over the past thirty years. In 2018, three West African countries had some of the highest child mortality rates worldwide: Nigeria, Mali, and Sierra Leone [1, 2]. The main causes of these deaths are pneumonia, complications of preterm birth, childbirth-related events, diarrhoea, and malaria [1]. These causes are exacerbated by malnutrition, affecting 6.9% of children under-five in West Africa by 2020 [3]. Most of these causes are treatable and preventable. Access to proper healthcare can significantly reduce disease-related morbidity and mortality by enabling early diagnosis, timely treatment, and complication prevention in children [4].

To address these access issues and bring healthcare services closer to the populations, WHO developed the “Global Strategy for Health for All by the Year 2000” [4]. Despite the inherent limitations of a pyramidal organizational approach [5], the focus has been on developing primary health care and structuring the public health system accordingly [6, 7]. Healthcare facilities range from small, decentralised primary healthcare centres (PHCs) to intermediate structures, such as district hospitals or regional hospitals, then national reference hospitals where specialised, highly technical care is provided [7]. The various structures are linked by a referral system organized according to the severity of the illness and the care capacities of the healthcare facilities. The private sector (e.g., approved or unapproved pharmacies, and private clinics) and traditional care complete the picture of the health system.

Despite efforts to bring healthcare closer to communities and implement reforms such as free healthcare, significant accessibility challenges persist, severely impacting vulnerable populations, especially children [8, 9]. However, there is limited information on how families navigate the health system before hospitalization. Most studies focus on parents' health-seeking behaviour for children who have died [10], specific diseases such as tuberculosis or pneumonia [11], or specific contexts [12]. They do not fully reconstruct children's actual care trajectories [13, 14]. Families' perceptions of PHCs, their reasons for choosing specific health services for their ill children, and the accessibility issues influencing their decisions of healthcare are key elements to consider. By analysing care pathways and their determinants, this study offers insights into how families navigate the health system—highlighting barriers, informing strategies to strengthen primary care and referrals, understanding alternative care-seeking behaviours, and guiding improvements in community-level prevention, timely care-seeking, and trust in PHCs.

This study is part of the AIRE project which aimed to enhance the detection of respiratory distress in children under-five by introducing a pulse oximeter (PO) during the Integrated Management of Childhood Illness consultations at PHC [15]. Early observations revealed a low incidence of severe cases at PHCs with variations across countries, suggesting that families might use alternative care pathways that bypass the PHCs [16]. Thus, this study aims to describe and measure the determinants of the care pathways of children aged 0–5 years presenting with severe illness at district hospitals in the four countries participating in the AIRE project (Burkina Faso, Guinea, Mali, and Niger).

## Methods

### Study design and sites

The ITINER’AIRE study is part of the AIRE research project carried out between 2020 and 2022, in Burkina Faso, Guinea, Mali, and Niger. The AIRE research protocol and details about the study locations have been previously published [15] (SF1). We conducted a descriptive cross-sectional study in seven district hospitals of the AIRE project (mainly located in rural districts), two per country, except for Guinea where only one hospital contributed [17]. The second district hospital in Niger were in urban area.

### Inclusion process

The study was implemented over one month (23 May—23 June 2022 in Burkina Faso, Guinea, and Mali, and 13 July – 12 August 2022 in Niger). All the children aged 0–5 years presenting at one of the seven district hospitals and classified by clinicians as severe cases were included after written parental consent.

Children were classified by clinicians at the triage level upon hospital admission (Emergency or Paediatric ward, depending on the country) based on the emergency triage form into three categories: urgent, priority, and ordinary [18]. We define as severe cases, children classified as urgent or priority. The triage form had previously been used by clinicians, except in Guinea, where it has been specifically introduced for the study.

Inclusion in the study was proposed to the families when the children were stabilised. After the child’s treatment has been established, the parental consent was required and then the interviews were conducted in a private, respectful setting by trained nurses already involved in the AIRE project. Particular attention was given to ensuring respectful and empathetic interactions to enhance the reliability and overall quality of the data.

### Data collection and main outcome

All data were collected using an interview (Supplementary file 2) developed for this study on a tablet-based electronic case report form (CRF) and stored on REDCap® software, with restricted access to guarantee data confidentiality. Any child visiting the hospital more than once during the month of data collection was considered as an independent event.

We collected socio-demographic characteristics of children and their caregivers to understand the family context in which children live (financial means available, caregivers' literacy, etc.). Moreover, we collected clinical data that highlighted the reasons why families sought care. The fever described in our study represents the threshold used by clinicians to identify children requiring urgent or priority attention. This criterion is met when the child's temperature exceeds 39.5°C in Burkina Faso and Niger, or 38.5°C in Guinea and Mali. The mid-upper arm circumference (MUAC), using WHO thresholds, estimated the prevalence of severe acute malnutrition (MUAC < 115 mm) and moderate acute malnutrition (MUAC between 115 and 125 mm) [19].

Care pathway since the onset of child’ disease was collected: we asked caregivers whether they had previously visited a traditional practitioner, a pharmacist, a community health worker, a health post, a private health centre, a primary health centre, a maternity ward (neonates), or a hospital. Pharmacist and private health centre regrouped under private care. We recorded the order of visits to the different facilities and the time elapsed since each visit to accurately reconstruct care pathways.

Information regarding obstacles to healthcare access and caregivers' perspectives on various health facilities was gathered to comprehend their care pathways. We used the framework of Levesque et al., presenting five dimensions of access to care, each of which has a demand side (patients) and a supply side (care system) [20]. These five dimensions are: 1) Approachability/ability to perceive 2) Acceptability/ability to seek 3) Availability and accommodation/ability to reach 4) Affordability/ability to pay 5) Appropriateness/ability to engage. We asked several questions on each of the five dimensions, using a Likert scale with five possible answers ranging from "strongly disagree" to "strongly agree" [21]. A few open-ended questions were also asked to understand care pathways, the responses were recorded by key words and then exploited according thematic groups.

In West Africa, the healthcare system follows a hierarchical structure known as the health pyramid, which outlines the recommended path for efficiently managing ill children. According to this pyramid, sick children typically begin their care pathway at a PHC, either referred by a community health worker or from a health post. If a child's condition is severe, they are then referred to a district hospital. Should additional care be necessary, they may be further referred to a regional, national or, university hospital level. In our study, “recommended" care pathways are those in compliance with this health pyramid. All children who consult other structures, such as pharmacists or traditional practitioners, are considered to be following an alternative pathway.

### Data analysis

Due to the varying contexts in each country regarding health services, the availability of health facilities, and health policies, we will present the outcomes for each country separately. In Niger, where one hospital was in a rural area, and the other in an urban area, the significant differences in pathway choice between these contexts have led to separate analyses.

We conducted descriptive analyses of the socio-demographic and clinical characteristics of children by country. We present the mean (standard deviation) and median (interquartile range) for quantitative variables, as well as frequencies and percentages for qualitative variables and thematic group.

A comparative analysis was conducted between the socio-demographic characteristics of children who visited a PHC and those who did not. A description of the care pathways followed by the children was done using Sankey diagrams [22]. We also described the delay of care according to whether the patient went to the PHC and according to the pathways recommended by the national health system. Spatial analysis produced a link map representing the relationships between the children's village of residence and the hospital. The relationships are also characterized by the average number of steps in a health system before reaching the hospital. This processing was carried out using the QGIS (3.24 Tisler version) geographic information system and the thematic cartography tools for processing extension to draw the links connecting the locations.

To determine the barriers and facilitators to access care according to Levesque's framework, we report the percentages of carers who agree or totally agree with the propositions. We used appropriate statistical tests to compare the responses relating to the PHCs and the hospital. Percentages were compared using Chi-2 tests or a Fisher exact test if appropriate. The significance level was set at 0.05 and all tests were two-tailed. All analyses were performed using R software, version 4.3.2 [23].

### Ethical considerations and consent to participate

The four national ethics committees (Burkina Faso n°2022/089/MS/MESRI/CERS, Guinea n°51/CNERS/22, Mali n°2022–068-MSDS-CNESS and Niger n° 038/2022/CNERS), the WHO Ethics Committee (n° ERC.0003788) have approved the protocol. The study has been conducted within the framework of the agreements signed with each hospital. After providing oral and written information about the project and, when necessary, involving a witness, written consent was obtained from the parents or the actual caregivers before registering any children. They were given a unique identifier associated with the data collected in order to guarantee confidentiality. The study was conducted in accordance with the ethical standards of these committees and with the 2013 Declaration of Helsinki and its later amendments.

## Results

**Fig. 1:**
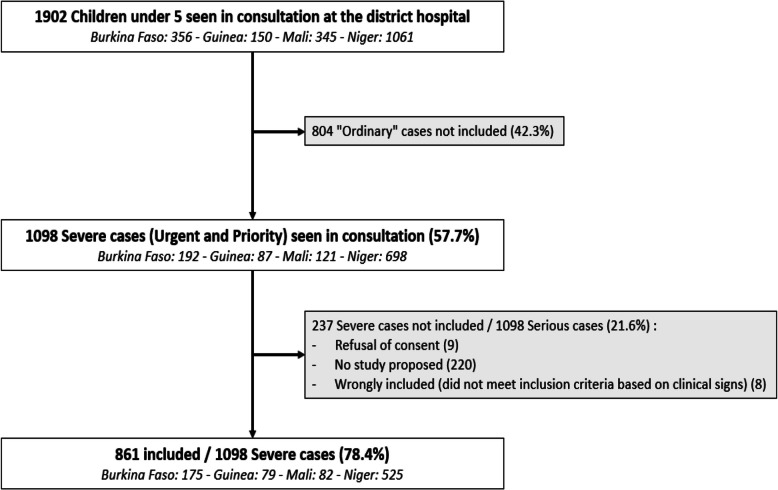
Flow chart of the ITINER’AIRE Study (Burkina Faso, Guinea, Mali, and Niger) (*N* = 861)

Over the study period, 1902 children under 5 years reached one of the seven district hospitals, of whom 1098 (57.7%) were defined as severe cases (Fig. 1). Among them, 861 children (78.4%) were included in this study: 175 (20.3%) in Burkina Faso, 79 (9.2%) in Guinea, 82 (9.5%) in Mali, and 525 (61.0%) in Niger (225 children in Dosso and 300 in Niamey).

The sociodemographic data of children and caregivers are outlined in Table 1. Overall, 52.4% of enrolled children were males, and 67.1% were aged between 2 and 59 months (Table 1). Mothers were the primary caregivers for the children, accounting for 92.7% in Niamey (Niger) and 68.0% in Burkina Faso. Regarding the education level of caregivers, 66.3% in Burkina Faso, 60.0% in Niger, 54.9% in Mali, and 49.4% in Guinea had no formal school education. Clinically, due to the study being conducted during the rainy season, children primarily presented at admission with respiratory symptoms (28.0%) or dehydration (25.7%) in Burkina Faso; dehydration and circulatory disorders (29.1% and 25.3%, respectively) in Guinea; and nutritional problems in Mali (31.7%). In Niger, dehydration was the most common presenting condition, affecting 58.7% of children in Niamey and 32.9% in Dosso.

**Table 1:** Socio-demographic and clinical characteristics of children included at hospital arrival, ITINER’AIRE study, 2022 (*N* = 861)

	**Burkina Faso *N* = 175**	**Guinea** ***N* = 79**	**Mali** ***N* = 82**	**Niger Niamey *N* = 300**	**Niger Dosso *N* = 225**
Socio-demographic data					
Child’s gender, male *N* (%)	110 (62.9)	56 (70.9)	48 (58.5)	179 (59.7)	118 (52.4)
Child’s age, *N* (%)					
Less than 2 months	61 (34,9)	12 (15.2)	32 (39.0)	29 (9.7)	74 (32.9)
2—59 months	114 (65.1)	67 (84.8)	50 (61.0)	271 (90.3)	151 (67.1)
Child's caregivers, *N* (%)					
Mother	119 (68.0)	49 (62.0)	41 (50.0)	278 (92.7)	158 (70.2)
Father	35 (20.0)	23 (29.1)	27 (32.9)	3 (1.0)	2 (0.9)
Other family members	21(12.0)	7 (8.9)	14 (17.1)	19 (6.3)	65 (28.9)
Caregiver's age, Med [Q1 -Q3]	29 [23,–36]	32 [27,–38]	30 [25,–38]	27 [23,–33]	30 [25,–40]
Missing, *N* (%)	0 (0)	3 (3.7)	0 (0)	0 (0)	0 (0)
Marital status, couple or married, *N* (%)	170 (97.1)	76 (96.2)	76 (92.7)	291 (97.0)	208 (92.4)
Level of education of caregiver, *N* (%)					
None	116 (66.3)	39 (49.4)	45 (54.9)	181 (60.3)	134 (59.6)
Primary school	24 (13.7)	6 (7.6)	25 (30.5)	48 (16.0)	28 (12.4)
Secondary school or university	35 (20.0)	34 (43.0)	12 (14.6)	71 (23.7)	63 (28.0)
Clinical data					
Malnutrition according to mid-upper arm circumference, *N* (%)					
Between 115 et 125 mm (MAM)	8 (4.6)	4 (5.1)	8 (9.8)	3 (1.0)	13 (5.8)
Under 115 mm (SAM)	11(6.3)	5(6.3)	11 (13.4)	9 (3.0)	37 (16.4)
Not applicable (children under 6 months)	78 (44.6)	18 (22.8)	39 (47.6)	49 (16.3)	94 (41.8)
Not measured	46 (26.3)	26 (32.9)	11 (13.4)	239 (79.7)	23 (10.2)
Fever, *N* (%)	77 (44.0)	41 (51.9)	29 (35.4)	97 (32.3)	45 (20.0)
Indications for hospitalisation, *N* (%)					
Respiratory disorders	49 (28.0)	6 (7.6)	22 (26.8)	29 (9.7)	42 (18.7)
Nutritional disorders	37 (21.1)	9 (11.4)	26 (31.7)	62 (20.7)	41 (18.2)
Neurological disorders	23 (13.1)	7 (8.9)	17 (20.7)	20 (6.7)	1 (0.4)
Circulatory disorders	29 (16.6)	20 (25.3)	11 (13.4)	17 (5.7)	38 (16.9)
Dehydration disorders	45 (25.7)	23 (29.1)	9 (11.0)	176 (58.7)	74 (32.9)
Trauma	5 (2.9)	1 (1.3)	-	-	-
Other disorders*	112 (64.0)	29 (36.7)	40 (48.8)	60 (20.0)	67 (29.8)
Outcome following consultation, *N* (%)					
Hospitalisation in this hospital	167 (95.4)	55 (69.6)	74 (90.2)	298 (99.3)	223 (99.1)
Transfer to a higher level	8 (4.6)	0 (0)	2 (2.4)	2 (0.7)	0 (0)
Return home proposed by a clinician	0 (0)	23 (29.1)	5 (6.1)	0 (0)	0 (0)
Return home against medical advice	0 (0)	0 (0)	1 (1.2)	0 (0)	0 (0)
Died on admission	0 (0)	1 (1.3)	0 (0)	0 (0)	2 (0.9)

*MAM* moderate acute malnutrition, *SAM* severe acute malnutrition

^*^Other signs included hypothermia, irritability, severe or intense pain, etc

### Care pathway for severe cases

**Fig. 2:**
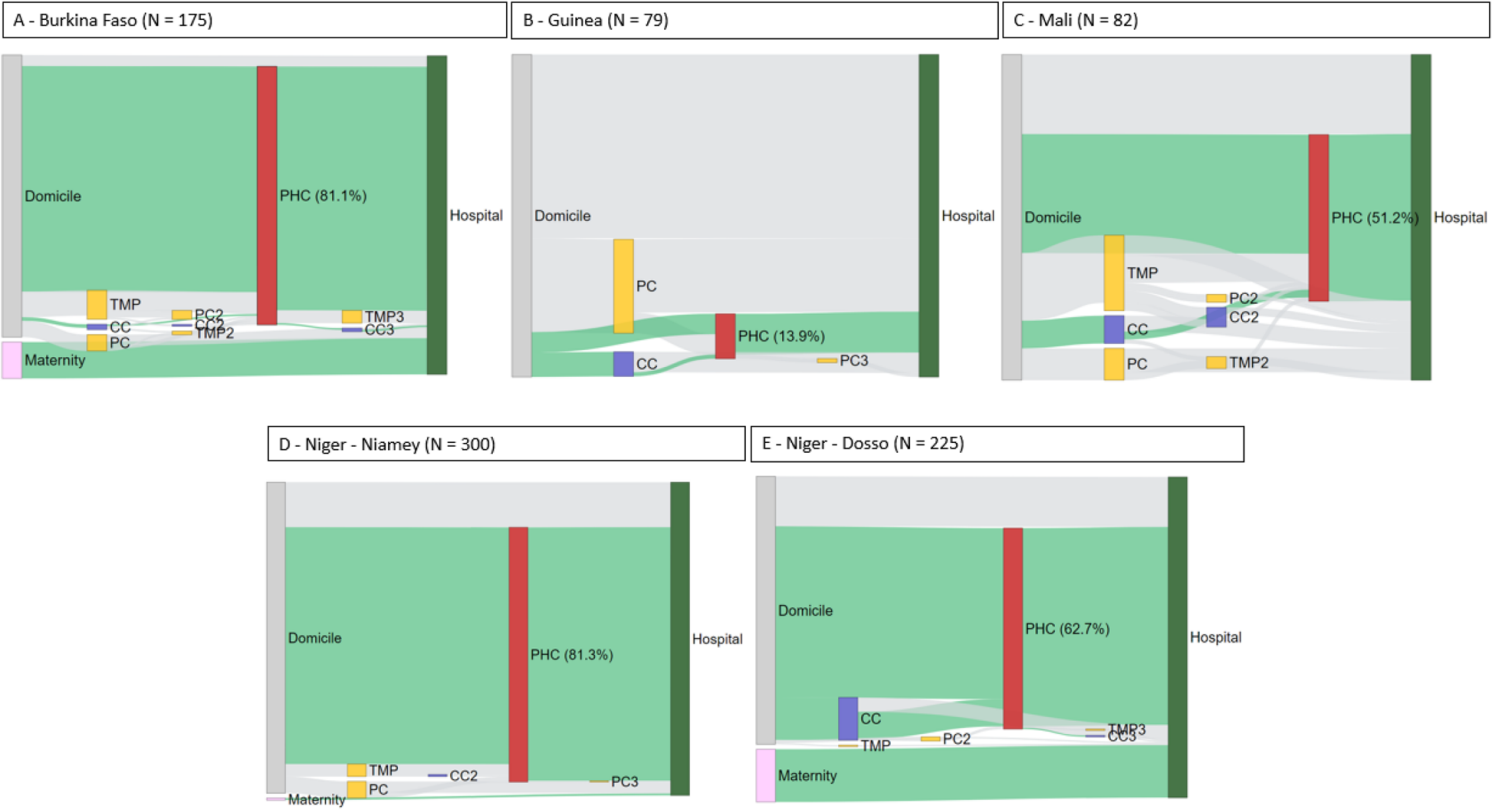
Sankey diagram illustrating the care pathways from domicile to the district hospital (*N *= 861). Legend: PC: Private care (pharmacist and private health centre), TMP: Traditional Medicine Practitioner, PHC: Primary Health Centre, CC: Community Care (community agent or health post). The numbers associated with the facilities have no meaning, solely for the construction of the graph. The "green" pathways align with the health system's recommendations

In Burkina Faso, most children (81.1%) were reported to have initially attended care at a PHC before being admitted to the district hospital (Fig. 2). The median duration between the onset of symptoms and the visit to the PHC was estimated at one day (SF 3). Only 3.4% of children went directly to the district hospital. Nearly 20.0% of children mentioned consulting a private structure before visiting the district hospital, including pharmacists, private health centres, or traditional medicine practitioners. Most children (> 70.0%) reported a traveling time of more than 30 min to reach the district hospital, and 72.5% reported traveling by private vehicle (motorbike or car) (Table 2).

**Table 2:** Description of facilities visited and children's care pathways, ITINER’AIRE study, 2022 (*N* = 861)

	Burkina Faso *N* = 175	Guinea *N* = 79	Mali *N* = 82	Niger Niamey *N* = 300	Niger Dosso *N* = 225
Structures visited during the care pathway, *N* (%)					
Primary Health Centre (PHC)	142 (81.1)	11 (13.9)	42 (51.2)	244 (81.3)	141 (62.7)
*PHC AIRE Research*	1 (0.7)	8 (72.7)	8 (19.0)	14 (5.7)	4 (2.8)
*PHC AIRE*	114 (80.3)	0 (0)	30 (71.4)	48 (19.7)	87 (61.7)
*PHC not included in AIRE*	27 (19.0)	3 (27.3)	3 (7.1)	182 (74.6)	50 (35.5)
*Missing*	0 (0)	0 (0)	1 (2.4)	0 (0)	0 (0)
Traditional medicine practitioner	23 (13.1)	0 (0)	21 (25.6)	12 (4.0)	2 (0.9)
Pharmacist	4 (2.3)	24 (30.4)	5 (6.1)	4 (1.3)	2 (0.9)
Private health centre	7 (4.0)	0 (0)	5 (6.1)	13 (4.3)	1 (0.4)
Community health worker	1 (0.6)	1 (1.3)	10 (12.2)	2 (0.7)	1 (0.4)
Health post	3 (1.7)	5 (6.3)	0 (0)	0 (0)	31 (13.8)
Hospital before inclusion	1 (0.6)	7 (8.9)	22 (26.8)	3 (1.0)	11 (4.9)
Maternity	20 (11.4)	0 (0)	0 (0)	2 (0.7)	37 (16.4)
Modalities of care pathway					
Number of steps with a health service before inclusion, *N* (%)					
Directly to hospital	6 (3.4)	45 (57.0)	20 (24.4)	43 (14.3)	35 (15.6)
1 step	142 (81.1)	28 (35.4)	45 (54.9)	239 (79.7)	167 (74.2)
2 steps	23 (13.1)	5 (6.3)	14 (17.1)	16 (5.3)	22 (9.8)
3 steps	4 (2.3)	1 (1.3)	3 (3.7)	2 (0.7)	1 (0.4)
Reported travel time to the hospital, *N* (%)					
*For children who went directly to the hospital (N)*	*6*	*45*	*20*	*43*	*35*
Less than 30 min	2 (33.3)	30 (66.7)	11 (55.0)	32 (74.4)	28 (80.0)
Between 30 min and 1 h	3 (50.0)	8 (17.8)	6 (30)	11 (25.6)	3 (8.6)
More than 1 h	1 (16.7)	7 (15.6)	3 (15.0)	0 (0)	4 (11.4)
*For children who consulted at least one facility (N)*	*169*	*34*	*62*	*257*	*190*
Less than 30 min	46 (27.2)	10 (29.4)	17 (27.4)	171 (66.5)	109 (57.4)
Between 30 min and 1 h	68 (40.2)	12 (35.3)	27 (43.5)	74 (28.8)	27 (14.2)
More than 1 h	55 (32.5)	12 (35.3)	18 (29.0)	11 (4.3)	54 (28.4)
Missing	0 (0)	0 (0)	0 (0)	1 (0.4)	0 (0)
Main modes of transport, *N* (%)					
Public ambulance or NGO	26 (14.9)	0 (0)	1 (1.2)	15 (5.0)	14 (6.2)
On foot	6 (3.4)	10 (12.7)	2 (2.4)	5 (1.7)	43 (19.1)
Taxi (car or motorbike)	3 (1.7)	66 (83.5)	3 (3.7)	235 (78.3)	148 (65.7)
Private vehicle (car or motorbike)	127 (72.5)	3 (3.7)	73 (89.0)	38 (12.6)	20 (8.8)
Autonomy over the choice of care pathway, *N* (%)					
Needed advice on the care pathway	35 (20.0)0	31 (39.2)	23 (28.0)	274 (91.6)	24 (10.7)
Person who has decided to seek the hospital					
Head of household	46 (26.3)	68 (86.1)	47 (57.3)	93 (31.0)	48 (21.3)
Other family members	10 (5.7)	2 (2.5)	6 (7.3)	9 (3.0)	18 (8.0)
Healthcare workers	119(68.0)	9 (11.4)	29 (35.4)	198 (66.0)	159 (70.7)
Medication received prior to hospital arrival, *N* (%)					
Pharmaceutical drugs	101 (57.7)	50 (63.3)	41 (50.0)	226 (75.3)	158 (70.2)
Pharmaceutical and traditional medicines	13 (7.4)	0 (0)	15(18.3)	3 (1.0)	0 (0)
Traditional treatments	1 (0.6)	2 (2.5)	7 (8.5)	3 (1.0)	2 (0.9)
No treatment	60 (34.3)	27 (34.2)	19 (23.2)	68 (22.7)	65 (28.9)

In contrast, in Guinea, a greater part of children (57.0%) indicated coming directly to the district hospital (Table 2 and Fig. 2), mainly on the advice of the head of the family (86.1%). Two-thirds of these children lived within 30 min of the district hospital. We observed that 13.9% of children sought consultation at a PHC, with a median delay of 5 days between the onset of symptoms and the PHC visit. At least 30.0% of children also consulted pharmacies (licensed or not), and more than 63% had taken medication before arriving at the hospital. Taxis were the most common means of getting to hospital for consultations (83.5%).

In Mali, 51.2% of children consulted first a PHC (Fig. 2, Table 2) with a median delay of 0 days between the onset of symptoms and the PHC visit (SF 3). One-fourth of the children (24.4%) bypassed PHC and went directly to the hospital, with 55.0% of them residing within a 30-min distance from the hospital. Multiple hospital visits were also observed: 26.8% of the children had already been to the hospital for the same symptoms (Table 2). Other healthcare workers were consulted more extensively compared to other countries, notably traditional medicine practitioners (25.6%), community health workers (12.2%), and private healthcare services (12.2%, including pharmacists and private health centres).

In Niger, most children sought consultation at PHCs before reaching the district hospital (73.3%). Besides examining care pathways, our selected sites allowed us to explore differences between urban (Niamey) and rural (Dosso) areas. Firstly, a higher proportion of children visited PHCs in urban areas compared to rural areas (81.3% vs. 62.7%, *p*-value < 0.001) (Fig. 2 and Table 2). Secondly, the analysis revealed that private healthcare workers (traditional medicine practitioners or private health centres) were more commonly utilized in urban areas than in rural areas (*p*-value < 0.05). Conversely, the use of community services was more prevalent in rural areas (*p*-value < 0.01). Lastly, in Niamey, accompanying mothers reported receiving more advice regarding the care pathway compared to Dosso (< 0.001), with the advice primarily coming from healthcare workers.

**Fig. 3:**
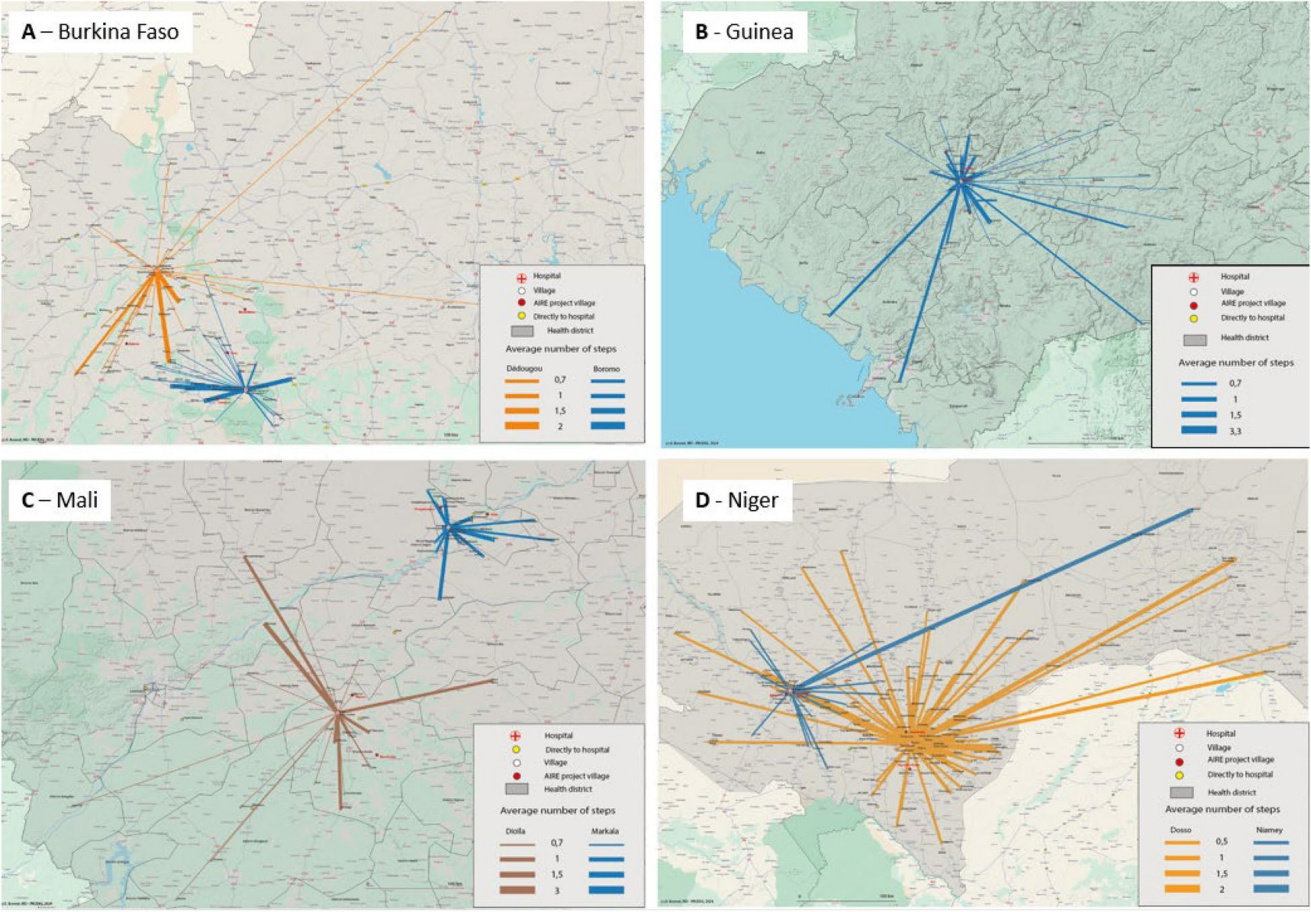
Geographic illustration of the origin of the patients and the average number of interactions with healthcare services. Made by children from each village before reaching the district hospital. The thickness of the lines indicates the number of steps taken. (ITINER'AIRE study, 2022; *N* = 861)

Figure 3 illustrates, for each country, a link between the children's village of residence and the hospital. Each link represents, by its length, the distance to the hospital, and by its thickness, the average number of steps taken in the health system before reaching the hospital. In most countries, the thickest links are as frequent as the thinnest, and there seems to be no link between the thickness of the line and the distance from the hospital.

### Determinants of care pathways

Across the countries, significant differences were observed in the socio-demographic characteristics of patients who visited a PHC compared to those who did not, particularly regarding the child’s age (Burkina Faso, Niger-Dosso), the caregiver’s identity (Burkina Faso, Mali, Niger-Dosso), and the caregiver’s education level (Mali, Niger-Niamey) (SF4). Patients who visited a PHC were generally more likely to be accompanied by their mothers and to have caregivers with lower levels of education, while children under 2 months were disproportionately represented among those who did not visit a PHC in certain countries (e.g., Niger-Dosso and Burkina Faso). However, no significant differences were found in patient care based on the child’s gender.

During the interviews, we found that the level of health literacy of families regarding the signs of simple and serious illnesses that they were aware of, varied considerably from one country to another. In Burkina Faso, for example, parents seem much better informed about how to distinguish a serious illness from a simple one. We also found that the power of the decision to seek care for a child depended very much on their father or the head of household. In Mali, for example, only 31.7% of the mothers could decide alone to bring their child to a health facility, compared to 92.6% for fathers.

In Burkina Faso, Guinea, and Niger-Dosso, caregivers tended to visit PHC more for mild illnesses over severe ones (SF 5). Mali and Niger-Niamey show no significant difference. Seeking directly hospital increased for serious illnesses in all countries, but the extent varies. In Burkina Faso, there was a notable disparity, with only 5% visiting hospitals for simple illnesses versus 96% for severe ones. In Guinea, most parents were likely to go to the hospital regardless of the illness severity (84% for simple and 100% for severe). In Mali, many opted for traditional practitioners (29%), or community health workers (23%) for simple issues, but primarily used PHC for severe conditions (66%), with a high resort to hospitals in the end (95%). In Niger's Niamey, PHC were popular for both simple and severe illnesses, with less use of hospitals for serious cases than in other health districts (74%). In Dosso, caregivers primarily used PHC for mild illnesses (96%), rarely hospitals (3%), and sometimes health posts (12%). For severe illnesses, they were least likely to use PHCs (32%) compared to other districts.

Levesque's framework enabled us to analyse the various dimensions of access to care for the different health structures and to compare caregivers’ perceptions of access between the PHC and the hospital (Table 3). Regarding the first dimension, respondents in Guinea expressed more confidence in hospitals than in PHC regarding improving their child's health (20% vs. 100%, *p*-value < 0.001). Across all countries, hospitals are preferred for serious illnesses (it’s particularly the case in Guinea where only 3% in PHC were confident that the care provided will improve their child's health if they are severely ill, vs 98% for the hospital, *p*-value < 0.001) and perceived to better meet health needs. According to the second dimension, few cultural or social barriers (discrimination, relationship problems, etc.) or barriers related to the acceptability of healthcare services were reported overall, but when differences between PHC and hospitals existed, hospitals were favoured. For dimension three, while PHCs were considered more accessible geographically (for example, 91% found the PHC easy to access from home vs. 39% for the hospital in Niamey, *p*-value < 0.001), hospitals were perceived to have better staff availability and opening hours in Mali and Niger. Except in Burkina Faso and in Guinea's hospital, waiting times for care are quite short. According to the respondents, medicines were perceived to be more available in hospitals, but, in dimension four, care was considered more affordable at PHC, except in Mali where it was roughly equivalent. This was particularly the case in Guinea, where caregivers were much less able to pay for healthcare costs in the hospital than in the PHC (87 vs. 12, *p*-value < 0.001). At the PHC, opportunity costs (travel time and impact on daily activities) were also considered to be lower than at hospitals in all countries. Finally, for dimension five, overall satisfaction and perceived quality of care favoured hospitals across all countries. Communication with health workers was generally good, with no significant differences between PHC and hospitals, except in Niger, where it seemed to be slightly better in hospitals.

**Table 3:** Percentage of caregivers who agree (agree or totally agree) with the questions based on the accessibility dimensions of the Levesque et al. framework (*N* = 861)

		***Burkina Faso (N* = *175)***			***Guinea (N* = *79)***			***Mali (N* = *82)***			***Niger—Niamey (N* = *300)***			***Niger—Dosso (N* = *225)***		
		PHC level	Hospital level	*p-value (1)*	PHC level	Hospital level	*p-value (1)*	PHC level	Hospital level	*p-value (1)*	PHC level	Hospital level	*p-value (1)*	PHC level	Hospital *level*	*p-value (1)*
Dimension 1: Accessibility/ability to perceive	Knowledge of available health care	81	65	***	93	100	*NS*	68	85	**	*53*	56	*NS*	81	94	***
	Confidence in the care provided to improve my child's health	91	98	**	20	100	***	68	98	***	76	96	***	80	99	***
	Confidence that the care provided will improve my child's health if he/she is severely ill	24	97	***	3	98	***	18	98	***	49	96	***	19	96	***
	Belief that the hospital better meets my needs	*97*			*100*			*92*			*88*			*98*		
Dimension 2: Acceptability/ability to seek	Equal service for everyone and no discrimination	94	96	*NS*	94	98	*NS*	68	86	***	78	83	**	81	87	*
	Presence of cultural barriers	6	2	*	5	2	*NS*	3	7	*NS*	2	0	*NS*	5	4	*NS*
	Acceptable health services	85	96	***	94	100	*NS*	73	97	***	87	98	***	88	98	***
	Good relations with health workers	85	83	*NS*	96	100	*NS*	69	73	*NS*	56	62	**	94	97	*NS*
Dimension 3: Availability and welcome/ability to seek	Easy to access from home	88	34	***	90	23	***	83	44	***	91	39	***	76	31	***
	Opening hours and staff availability are sufficient	97	99	*NS*	95	40	***	65	93	***	89	95	***	82	91	***
	Long waiting times	35	25	*NS*	2	90	***	5	6	*NS*	5	7	*NS*	16	15	*NS*
	Availability of drugs	43	78	***	91	99	*NS*	43	88	***	73	90	***	60	79	***
Dimension 4: Affordability/ability to pay	Hospital is less expensive than PHC	23			10			52			35			11		
	Lower opportunity costs to seek care in PHC than in hospital	79			89			65			94			87		
	Availability of financial means to pay for care	27	20	*	87	13	***	66	70	*NS*	75	70	*	78	62	***
	Ability to pay for non-medical costs	29	34	*NS*	4	4	*NS*	65	48	*	75	69	*	88	78	***
Dimension 5: Appropriateness/ability to engage	Good perceived quality of care received	92	99	***	37	96	***	65	94	***	79	99	***	84	99	***
	Satisfaction with care received	85	97	***	29	100	***	60	90	***	77	99	***	84	98	***
	Ease of communicating with health workers	94	97	*NS*	96	100	*NS*	68	82	*NS*	80	87	***	92	96	*
	Good understanding of recommendations received from health workers and ability to apply them	100	99	*NS*	97	99	*NS*	69	90	***	93	99	***	96	99	*

(1) *p < 0,05, **p < 0,01, ***p < 0,001, ns = non significant

## Discussion

To our knowledge, this is the first study to analyse care pathways for critically ill children under five across four West African countries with diverse health system structures and sociocultural contexts, at the same time. This multicountry approach provides a unique opportunity to capture a wide range of real-life care trajectories, offering valuable insights into how families navigate referral systems in low-resource settings. While existing literature often focuses on parental care-seeking behaviour in cases where the child has died [10], or on specific diseases such as tuberculosis or pneumonia [11], or particular national contexts [12], few studies have attempted to reconstruct the full care trajectories of children across multiple countries. Our findings fill this gap by highlighting the complexity and variability of referral patterns, and health system responses across settings.

Using data collected from caregivers and spatial analysis, we examined the care trajectories of 861 children admitted to district hospitals, comparing them to recommended public health pathways. Our findings reveal a wide variety of routes taken by families—often combining public system structures (health posts, PHCs, district hospitals) with parallel care options such as traditional healers, private clinics, and pharmacies. In Burkina Faso and Niger, adherence to the recommended pathway—consulting a PHC before hospital admission—is relatively high. In contrast, only 15% of children in Guinea visited a PHC prior to hospitalization. Traditional healers play a significant role in Mali, while private care providers are more frequently consulted in Guinea than in other countries. In Niger, we also observed contrasting care-seeking behaviours between urban Niamey and rural Dosso, largely driven by differences in healthcare availability. Levesque's framework offered a strong conceptual foundation for designing our data collection tools and analysing barriers to healthcare access. We closely examined factors such as facility availability, care services, geographic distance, affordability, and the child’s clinical condition. The findings provided valuable insights into how parents make decisions regarding specific care pathways.

We will explore and try to explain the key determinants of the care pathways identified. The diverse routes taken by families are influenced by several factors, including the service availability and cost, the context of illness management, and barriers to accessing care.

The first determinant examined is how the cost of services influences care pathways. Burkina Faso and Niger are the two countries with the highest proportions of direct pathways to PHCs. These findings are supported by a study in Burkina Faso (2021), where more than 80% of respondents who were ill in the 15 days before the survey reported visiting PHC, and similar patterns were also observed in Niger [24, 25]. Since 2006 in Niger and 2016 in Burkina Faso, governments have implemented free healthcare programs for children under-five and pregnant women to improve access and encourage the use of health services [9, 26]. By improving financial accessibility, these reforms encourage families to visit PHCs and seek care earlier, while limiting costs [27, 28]. In West Africa, several studies have demonstrated the benefits of these policies in increasing the use of health services for children [26, 29–31]. In Guinea and Mali, healthcare policies—considered "partial"—provide free services only for a limited number of diseases, such as HIV, tuberculosis, malaria, and malnutrition. As a result, families may turn to alternative providers, including traditional healers, pharmacists, or drug vendors, which are often perceived as more affordable. We also observe that caregivers, particularly those from wealthier households, tend to bypass intermediary healthcare structures and go directly to hospitals, as also suggested by by Zeng et al. in Zimbabwe [32]. This trend can be attributed to several factors: shorter distances to hospitals, which minimize both travel costs and steps; higher hospital fees, which may be offset by the perceived quality of care; and frequent drug shortages at PHCs. Only 52% of essential child health medicines were available and not expired in healthcare facilities in Niger in 2019 [33]. Additionally, drugs in private pharmacies are also costly. Families choose their care pathways based on the resources available to them. Wealthier households tend to opt for more expensive care, perceived as a guarantee of quality, while lower-income families must navigate between the nearest and most affordable facilities to minimize direct and indirect costs related to travel and/or time off work. In this way, the cost of care significantly influences care pathways.

In the following section, we will explore the second determinant: the availability of healthcare services, which also plays a significant role [34]. The availability of healthcare vary not only between countries but also within them [33, 35]. In Niger, the density of healthcare facilities per 10,000 inhabitants is 1.6 in Dosso (rural) and 2.5 in Niamey (Urban) [33]. Hence, limited access to services may lead parents to choose more accessible or familiar options, even if they are not necessarily the most suitable for their child's condition. Hospitals and PHCs are more commonly found in urban areas, where demand is highest, along with private facilities such as pharmacies and private clinics. In contrast, in rural areas, to compensate for the lack of healthcare services, alternatives such as community agent or health posts have been established. As a result, families in these areas tend to rely more on nearby healthcare services, leading to differences in care pathways [36]. Traditional medicine, including herbalists and religious healers, is often one of the main health care options for some rural households. As noted by Scott et al., their use depends on the nature of the illness (e.g., seizures, sunken fontanel), limited access to modern care, cost, and social connections [37, 38]. Many parents relied on traditional healers due to financial and geographical barriers, a trend particularly evident in rural Mali in our study and also reported in the literature in other countries [39–41].

In addition to facility availability, geographical access significantly influences care pathways. In rural and remote areas, factors such as poor roads, mountains, rivers, limited transport, and uneven population distribution hinder healthcare access. For example, the 2018 SARA survey in Burkina Faso showed an average distance of 6.4 km to basic healthcare in Boucle du Mouhoun, compared to 1.6 km in the central region [42]. Our study also found that, except in urban Niamey, about one-third of families travelled over an hour to reach the hospital. However, in Guinea, two-thirds of families within 30 min of a hospital still chose direct hospital visits instead of local PHCs, reflecting a preference linked to perceived care quality [37, 43]. A Malian study by Treleaven et al. found that children living within 2 km of a health centre were twice as likely to consult a qualified provider within 24 h compared to those living 2–5 km away [44]. Despite geographic challenges, some families optimize their travel routes, while others, lacking access to certain facilities, rely on the nearest providers (Fig. 3).

The perceived severity of the condition plays also a significant role in shaping care pathway decisions. Parents frequently choose healthcare facilities based on their own assessment of their child's condition, often opting for direct hospital attendance in cases they consider severe, despite recommended pathways suggesting alternative options (PHCs). This highlights the challenge of recognizing the severity of children's illnesses. Our study found varying levels of awareness across countries, and a 2014 systematic review by Geldsetzer et al. showed that families generally struggle to recognize the severity of conditions like diarrhoea, malaria, and pneumonia [44]. In Burkina Faso, the ITINER’AIRE study demonstrated notably higher recognition, likely reflecting the success of recent community health initiatives. This lack of knowledge can delay care or influence care pathways. However, the literature suggests that the relationship between education level and adherence to care pathways remains unclear [25].

Gender dynamics significantly impact access to child healthcare services, with many mothers reliant on their husbands for financial resources and transportation, resulting in limited autonomy in decision-making regarding their children's treatment and care pathways. This phenomenon is extensively documented in the literature [43, 45, 46].

Finally, another factor is the variation in how each country implements the care pyramid. In Burkina Faso, compliance with the pyramid is enforced by requiring written proof of referral from the primary healthcare centre upon arrival at the hospital. In contrast, the care pyramid may be less familiar in other countries, and non-compliance may not necessarily result in reprimand.

Through this study, we highlighted that the healthcare pathways chosen by families for their children depend on several factors, including economic, accessibility, and medical considerations.

Based on Levesque’s framework, we also investigate how families choose care pathways according to their perception of healthcare facilities. Across all surveyed countries, families generally perceive hospitals as providing superior care compared to PHCs. This preference is driven by the perceived higher quality of care, trust in institutional capabilities, and better availability of equipment, qualified staff, and treatment options in hospitals – perceptions confirmed by national surveys showing that hospitals are better equipped and staffed than PHCs [33, 42]. Similar findings from studies in Sierra Leone highlight concerns about PHC challenges, including limited staff, long wait times, medicine shortages, and perceived inadequate care [37]. In our study, in Burkina Faso and Niger, despite the high perception of hospital care, families choose PHCs first due to factors previously mentioned, such as lower costs with free healthcare policies. In contrast, in Guinea, hospitals are perceived as offering better care than PHCs, and families tend to bypass PHCs entirely, opting for direct hospital visits despite higher costs and longer travel distances, to secure the best outcomes for their children. This highlights the necessity for enhancing primary healthcare facilities to instil trust and ensure that high-quality, accessible care is universally available throughout the healthcare system.

In the end, while representations of care pathways are often linear, this is rarely the case. Care pathways are non-linear, with families combining care opportunities for their children as best as they can, depending on the assessment of the type of sickness, the course of the sickness, and their available means to invest in treatment [47]. Thus, they navigate the healthcare system between their capabilities, barriers to accessing care, and their child's condition. Pathways can be concomitant (using multiple types of care simultaneously), interrupted (if the child's condition improves or financial resources are insufficient), and evolving. Caregivers’ care-seeking decisions are influenced by a wide range of factors that are difficult to isolate or prioritize, combining both objective constraints and subjective perceptions of the available options.

Our study presents several limitations to consider. Initially, we aimed to collect comprehensive data collection to accurately represent care pathways for critically ill children under-five presenting at hospital but some factors may affect the representativeness of our sample. First, the sample's adequacy in representing the region and country is uncertain, as we included only two health districts per country, mainly rural (except in Niger) and only one hospital in Guinea. Second, although the chosen hospitals function as district referral hospitals, sometimes hospitals in neighbouring districts are closer to certain areas on the outskirts. Thus, families facing health problems may prefer to go to these neighbouring hospitals rather than to the district referral hospital (e.g., certain regions of Guinea and Mali). Third, the classification and selection of severe cases vary slightly between countries in terms of definitions and remain subject to clinician judgment, introducing a degree of heterogeneity that could affect cross-country comparability. As previously mentioned, many critically ill children may have been excluded from the study, as they never reached the hospital due to a lack of resources. Fourth, as the data was collected during a single month of the year, this does not allow us to illustrate the variations in care pathways according to seasonal factors (seasonal pathologies, rainy seasons, etc.). Then, despite exhaustive data collection efforts, few children may have been missed. Some of the data, especially clinical information, relied on hospital medical registers, which are contingent on thorough completion by healthcare personnel, a practice that may not always be consistent. Moreover, some data, particularly regarding the onset of initial symptoms, may be subject to recall bias.

Finally, interviewing caregivers at the hospital may have influenced their responses due to social desirability bias and concerns about affecting their child's care. They might have been reluctant to mention informal healthcare use or express negative views on hospitals. Lastly, interviewing caregivers of critically ill children could have affected their mental state and focus, as they were understandably preoccupied with their child's health while awaiting stabilization.

In terms of perspectives, future studies should incorporate qualitative interviews to gain a deeper understanding of non-financial barriers and to better assess the perceptions of both clinicians and parents. Analysing longitudinal data would enable us to describe the influence of healthcare pathways on disease progression, particularly due to therapeutic missteps (delayed treatment initiation, seeking consultation outside recommended facilities).

Our findings point to several key interventions to strengthen referral pathways for critically ill children [48]. First, community sensitization is essential to improve caregivers’ understanding of illness severity and the urgency of timely consultation. Second, enhancing the quality of care at primary healthcare facilities is critical to support early recognition and initial management of severe cases, thereby reinforcing reliability in cares at PHC, reducing delays and minimizing the bypassing of PHCs. Third, there is a need to clarify and operationalize referral policies, including improving communication between different levels of the health system and removing financial and logistical barriers—such as the lack of reliable transport—which currently hinder effective referrals, particularly over long distances.

## Conclusion

Our study aimed to explore and assess the factors influencing the care pathways of children aged 0–5 years with severe illness across various districts in West African countries. In countries where free healthcare policies are implemented, most children follow the recommended care pathway by initially seeking care at PHCs before hospital admission. Furthermore, our findings suggest the need for interventions aimed at improving geographical, equitable, and financial accessibility, as well as enhancing caregivers' trust in healthcare facilities, particularly PHCs. Enhancing the quality of care and reception at PHCs, along with improving perceptions of these facilities, can encourage families to seek care closer to home, and at an earlier stage, thereby reducing the burden of childhood illnesses.

## Supplementary Information

Supplementary Material 1. Supplementary file 1: Main characteristics of the district hospitals including in the ITINER'AIRE Study, 2022. Supplementary file 2: ITINER'AIRE survey 2022 form. Supplementary file 3: Time (in days) between first symptoms and arrival at the hospital according to visit or not to a PHC (A) and according to the recommendations of the healthcare system (B), ITINER'AIRE (*N* = 861). Supplementary File 4: Comparisons of the socio-demographic characteristics of patients according their attendance or not a PHC by countries. Supplementary file 5: Structures visited by children according to whether the illness is perceived by parents as simple or severe, ITINER'AIRE Study 2022 (*N* = 861).

## Data Availability

The datasets generated and analysed during the current study are not publicly available. Access to processed deidentified participant data will be made available to any third Party after the publication of the main AIRE results stated in the Pan African Clinical Trial Registry Study statement (PACTR202206525204526, registered on 06/15/2022), upon a motivated request (concept sheet), and after the written consent of the AIRE research coordinator (Valeriane Leroy, Valeriane.Leroy@inserm.fr, Inserm U1295 Toulouse, France, orcid.org/0000–0003-3542–8616) obtained after the approval of the AIRE publication committee, if still active.
