# Supplementary material for: Care pathways for critically ill children aged 0–5 years arriving at district hospitals in Burkina Faso, Guinea, Mali, and Niger (2022): a cross-sectional study
Source: BMC Public Health. 2025 Nov 13;25:3934. doi: 10.1186/s12889-025-24835-1 (PMC12613918; doi:10.1186/s12889-025-24835-1)
Supplement: Supplementary file 1 — Supplementary Material 1. Supplementary file 1: Main characteristics of the district hospitals including in the ITINER'AIRE Study, 2022. Supplementary file 2: ITINER'AIRE survey 2022 form. Supplementary file 3: Time (in days) between first symptoms and arrival at the hospital according to visit or not to a PHC (A) and according to the recommendations of the healthcare system (B), ITINER'AIRE (N = 861). Supplementary File 4: Comparisons of the socio-demographic characteristics of patients according their attendance or not a PHC by countries. Supplementary file 5: Structures visited by children according to whether the illness is perceived by parents as simple or severe, ITINER'AIRE Study 2022 (N = 861). [file 12889_2025_24835_MOESM1_ESM.docx]

Supplementary Files

[Supplementary file 1: Main characteristics of the district hospitals including in the ITINER'AIRE Study, 2022. 2](#_Toc203485059)

[Supplementary file 2: ITINER'AIRE survey 2022 form 3](#_Toc203485060)

[Supplementary file 3: Time (in days) between first symptoms and arrival at the hospital according to visit or not to a PHC (A) and according to the recommendations of the healthcare system (B), ITINER'AIRE (N = 861). 14](#_Toc203485061)

[Supplementary File 4: Comparisons of the socio-demographic characteristics of patients according their attendance or not a PHC by countries. 15](#_Toc203485062)

[Supplementary file 5: Structures visited by children according to whether the illness is perceived by parents as simple or severe, ITINER'AIRE Study 2022 (N = 861) 17](#_Toc203485063)

# *Supplementary file 1: Main characteristics of the district hospitals including in the ITINER'AIRE Study, 2022.*

|  | **Burkina Faso** | | | **Guinea** | **Mali** | | | **Niger** | |
| --- | --- | --- | --- | --- | --- | --- | --- | --- | --- |
|  | Boromo | Dédougou | Télimélé | | Dioila | Markala | Dosso | | Niamey |
| **Rural district** | Yes | Yes | Yes | | Yes | Yes | Yes | | No |
| **Health services available** |  |  |  | |  |  |  | |  |
| Pediatrics | ✓ | ✓ | ✓ | | ✓ | ✓ | ✓ | | ✓ |
| Noenatology | × | ✓ | × | | ✓ | × | ✓ | | ✓ |
| Pediatric emergencies | × | ✓ | ✓ | | ✓ | ✓ | ✓ | | ✓ |
| General consultation | ✓ | ✓ | ✓ | | ✓ | ✓ | ✓ | | ✓ |
| Hospitalisation | ✓ | ✓ | ✓ | | ✓ | ✓ | ✓ | | ✓ |
| Nutrition CRENI | ✓ | ✓ | ✓ | | ✓ | ✓ | ✓ | | ✓ |
| **Health professionals** |  |  |  | |  |  |  | |  |
| General practitioner | 1 | 4 | 2 | | 5 | 3 | 0 | | 1 |
| Pediatrician | 0 | 4 | 0 | | 0 | 0 | 4 | | 5 |
| Nurse | 3 | 34 | 5 | | 8 | 3 | 12 | | 10 |
| Midwife | 0 | 9 | 7 | | 3 | 7 | 1 | | 5 |
| Biomedical technician | ✓ | ✓ | ✓ | | ✓ |  | ✓ | | ✓ |
| **Free health care policy** | Full | Full | Partial | | Partial | Partial | Full | | Full |
| **Cost of a consultation for a child under 5 in XOF at HD** | 0 (0) | 0 (0) | 310 (0,5) | | 620 (1,1) | 1000 (1,7) | 500 (0,9) | | 0 (0) |
| **Average cost of an IMCI consultation in XOF at the PHC** | 0 (0) | 0 (0) | 250 (0,4) | | 584 (1) | 468 (0,8) | 0 (0) | | 0 (0) |

# Supplementary file 2: ITINER'AIRE survey 2022 form

**ITINER'AIRE SURVEY**

Care pathway for "severe cases" of children aged 0-5 years arriving at AIRE project district hospitals (Burkina Faso, Guinea, Mali and Niger)

**PART I: Interview with the child's family or accompanying person following the consultation**

Consultation date: |__|__| / |__|__| / 2022

Child's condition (triage form): **□ URGENT CASE □ PRIORITY CASE □ SIMPLE OR ORDINARY CASE**

**GUINEA**: Child's condition (triage form): **□ SEVERE CASE □ SIMPLE CASE**

Consent: □ Accepted □ Refused, reason: ...............................................................................................

**Socio-demographic data of the child**

Sex: □ Male □ Female

Date of birth: |__|__| / |__|__| / |__|__|__| or age: |__|__|

Origin (Village): ..............................

Prefecture (or district) :

| ***Burkina Faso*** | ***Guinea*** | ***Mali*** | ***Niger*** |
| --- | --- | --- | --- |
| □ Dédougou  □ Boromo  □ Other: ........................... | □ Télimélé  □ Other: ........................... | □ Dioila  □ Markala  □ Other: ........................ | □ Niamey IV  □ Dosso  □ Other: ........................... |

**Socio-demographic data on the carer**

Who is the accompanying person on this day?

□ Mother □ Father □ Aunt, uncle □ Grandparents □ Brother, sister □ Other family member

□ Other: ........................

Age of accompanying person: |__|__| years

What is his family situation?

□ Currently married/coupled □ Single □ Divorced □ Widowed □ Refused

The accompanying person (in any language) :

□ Can read or write

□ Can't read or write

What is the educational level of the carer?

□ None □ Primary school □ Secondary school □ University study

**The child's care pathway before hospital admission**

Reassure the family that you are not there to judge the answers. They can answer the questions freely, it will not affect the care of their children.

How many days ago did the first signs of illness begin? |__|__| days

*Has the child ever consulted a doctor before today for this disease (since the 1^ers^ signs)?*

Has the child consulted **a healer, traditional healer or marabout** since the onset of symptoms ?

□ Yes □ No □ Don't know

Has the child been to **a pharmacist or drug seller** since the onset of symptoms?

□ Yes □ No □ Don't know

Has the child seen **a community health worker or a community health link** since the onset of symptoms?

□ Yes □ No □ Don't know

Has the child been to **a health post** since the onset of symptoms?

□ Yes □ No □ Don't know

Has the child been to a **private health center** since the onset of symptoms?

□ Yes □ No □ Don't know

In which private centre? □ Health care practice □ Private clinic

Has the child been to **hospital** since the onset of symptoms (or hospitalisation)?

□ Yes □ No □ Don't know

In which hospital? ......................................

Has the child visited **a primary health centre (PHC)** since the onset of symptoms?

□ Yes □ No □ Don't know

In which health centre? ......................................

*If the PHC ticked is one of the AIRE Research PHCs:* Has your child been included in the AIRE research project? □ Yes □ No □ Don't know

*Recall the purpose of the AIRE project and how it works. Ask if the family has the AIRE card with them or try to find the identifier at hospital or PHC level.*

If so, what is its identifier? ID AIRE |__|-|__|__|-|__|__|__|__|

Has the child been to any other **health facility or person** not mentioned above?

□ Yes □ No □ Don't know

Specify:........................................................

**SUMMARY TABLE OF THE COURSE**

*Tick only the facilities consulted during the home-hospital journey (previous answers)*

| **Structures consulted** | **How many days ago?** | **Order** |
| --- | --- | --- |
| □ Healer, tradipratician, marabout | \|__\|__\| | \|__\| |
| □ Pharmacist or drug seller | \|__\|__\| | \|__\| |
| □ Community agent or relay | \|__\|__\| | \|__\| |
| □ Health post | \|__\|__\| | \|__\| |
| □ Private health centre | \|__\|__\| | \|__\| |
| □ Hospital | \|__\|__\| | \|__\| |
| □ Primary health centre | \|__\|__\| | \|__\| |
| □ Other | \|__\|__\| | \|__\| |

Has the child ever been given medication for symptoms before arriving at the hospital?

□ No medication

□ Traditional medicines

□ Pharmaceutical drugs

□ Traditional and pharmaceutical medicines

□ Don't know

If yes, who advised these medicines (Several choices possible)?

□ A health professional

□ You, your family or friends

□ A drug seller

□ A traditional practitioner or healer

□ Other: .....................................

Who made the initial decision to send your child to hospital?

□ Head of household

□ Other family members

□ Community health worker or community liaison officer

□ Clinician in a health facility

□ Other: ............................................

What means of transport did you use to come to the hospital?

| □ Public ambulance  □ Private car  □ Bicycle  □ On foot | □ private ambulance  □ Bus  □ Private motorcycle/tricycle  □ Other, specify:................ | □ Car Taxi  □ Charette  □ Motorcycle/tricycle taxi |
| --- | --- | --- |

How long did it take you to get to the hospital?

*For this question, count the travel time from home to the hospital, if the mother made a stop , do not count the time of the stop.*

□ Less than 30 min

□ Between 30 minutes and 1 hour

□ Between 1 and 2 hours

□ More than 2 hours

**Oxygen therapy**

Are you familiar with the pulse oximeter ?

*Explain the use of the PO and show the device or a picture of the device.*

□ Yes □ No

If so, has this changed your choices about your child's health pathway?

□ Yes □ No □ Don't know

If so, in what way?

|  |
| --- |

Was an oxygen saturation measurement taken before arrival at the hospital?

□ Yes □ No □ Don't know

If yes, location of measurement

□ Pharmacist or drug seller

□ Health worker or community relay (off-site)

□ Health post

□ Consultation in a primary health centre

□ Hospital consultation or hospitalisation

□ Consultation of a private structure

□ Other, specify:...............................................

And oxygen saturation value: |__|__|__| % □ Do not know

Was oxygen therapy started before arrival at the hospital?

□ Yes □ No □ Don't know

| **PART II: To be completed using the triage form, clinician's log, and medical record** |
| --- |

**Clinical data on admission to hospital**

Weight: |__|__| kg|__|__| g □ Not measured

Size: |__|__|__|cm □ Not measured

Brachial circumference (from 6 months): |__|__|__|mm □ Not measured

What sign or signs led to the classification as a **severe case**?

**Multiple choice:** *to be ticked according to the signs ticked on the triage form.*

| *Disorders*  *respiratory* | *□ Obstruction*  *□ Suffocation*  *□ Central cyanosis*  *□ Not breathing or no breathing*  *□ Severe respiratory distress*  *□ Noisy breathing*  *□ Difficulty or distress in breathing* |
| --- | --- |
| *Nutrition* | *□ Brachial circumference < 110 mm*  *□ Visible and severe weight loss*  *□ Edema of the feet or Edema of both feet*  *□ MAS + 1 priority sign*  *□ MAS new admission* |
| *Traffic* | *□ Shock, traffic problem*  *□ Cold extremities or cold hands*  *□ Skin recolouration time (CRT) > 3 sec*  *□ Pulse rapid, weak*  *□ Intense pallor*  *□ Severe palmar pallor* |
| *Neurology* | *□ Coma*  *□ Convulsion*  *□ Consciousness disorders (lethargy, hypotonia, agitation)*  *□ Confusion* |
| *Dehydration* | *□ Severe dehydration*  *□ Lethargy*  *□ Hollow or sunken eyes*  *□ Skin fold (> 3 or > 2 sec)*  *□ Unable to drink*  *□ Diarrhea*  *□ Vomiting*  *□ Vomits everything he consumes* |
| *Trauma* | *□ Trauma or severe trauma*  *□ Surgical case, recent surgery*  *□ Burns* |
| *Temperature* | *□ Hypothermia (T° < 35 °C)*  *□ Fever (≥39°5 C or >38°5)* |
| *Other* | *□ Severe hypoglycaemia (< 54 mg/dl)*  *□ Sick child/infant under 2 months*  *□ Continued instability, irritability or agitation*  *□ Severe or severe pain*  *□ Intoxication, poisoning*  *□ Emergency referral*  *□ Other, please specify: .............................................................* |

Type of "serious case"? *(If a sign is ticked under 'Respiratory disorders' or other respiratory signs in the medical record*)

□ Respiratory

For which respiratory sign? .............................................................................

□ Non-breathing

**Regarding the measurement of oxygen saturation :**

Was an oxygen saturation measurement taken on arrival at the hospital?

□ Yes □ No □ Don't know

*If yes :*

Oxygen saturation value: |__|__|__| % □ Not measurable

Heart rate: |__|__|| per min □ Not measurable

If saturation < 90%, was oxygen therapy started in hospital?

□ Yes □ No □ Don't know

If not, why not? ...........................................

**PART III: Decision following consultation with the child**

What happens to the child after the consultation?

□ Hospitalization in this hospital

□ Transfer to a higher level

□ Return home proposed by the clinician

□ Return home against medical advice

□ Deceased

□ Other, please specify: ..........................................................

**PART IV: Exploring pathway choices**

**Have you sought advice on where to take your child for treatment?**

□ Yes □ No

If yes, to whom (depending on the relationship of the person to the carer)

□ Spouse □ Parents □ Grandparents □ Brother, sister □ Other family members

□ Friends □ Other: ........................

**Describe how you distinguish between your child having a simple disease and a severe disease (*open question, keywords)***

|  |
| --- |

**We will now ask you some questions to find out, when your child is ill, who you usually take them to see.**

*Interviewer's note: Emphasise that you want to know the usual route, which may be different from the route taken today.*

*(Several answers are possible, please indicate the precise order)*

When you think it is a simple disease (parent's perception), you take it away:

□ Healer, tradipratician or marabout

□ Community health worker/community relay

□ Private centre

□ Pharmacist or drug seller

□ Health post

□ CSP

□ District Hospital (HD)

□ Other, specify: ........................

When you think it is a serious illness (parent's perception), you take it away:

□ Healer, tradipratician or marabout

□ Community health worker/community relay

□ Private centre

□ Pharmacist or drug seller

□ Health post

□ CSP

□ District Hospital (HD)

□ Other, specify: ........................

***Question for the interviewer: does the route of the day correspond to the usual route followed by the child in case of serious illness?* □ Yes □ No**

**If not, ask why this pathway has changed today *(open question, keywords)***

|  |
| --- |

The questions in this section will assess your level of agreement or disagreement with each of the following statements. Please tick only one answer per question. The scale used is a graded scale with 5 answers to qualify the degree of agreement. For example:

**1. Strongly agree:** means full agreement

**2. Agreed**: means simply agreed

**3. Neither disagree nor agree**: if you have no opinion on the issue (neutral)

**4. No agreement**: means simple disagreement

**5. Strongly disagree**: to express total disagreement.

The questions will be adapted, following an algorithm that will include the answers given by the person regarding the usual pathway that he/she has for his/her child (in case of serious and non serious illness). In the case where the person has only one step in their usual care pathway (e.g. only HD), we will still maintain a comparison between the CSP and the HD (District Hospital) in the questions. *In other cases, each care "potential" will be proposed in the framework of each question*.

Example: if the person's usual care pathway is ASC - CSP - HD, then the questions would be of this form:

I am generally satisfied with the care my child receives when I go for a consultation...

- A CSA: □ Strongly disagree □ Disagree □ Neither disagree nor agree □ Agree □ Strongly agree
- To the PSC : □ Strongly disagree □ Neither disagree nor agree □ Agree □ Strongly agree
- To the HD: □ Strongly disagree □ Neither disagree nor agree □ Agree □ Strongly agree

|  | **Strongly disagree** | **Disagree** | **Neither disagree nor agree** | **Agree** | **Strongly agree** |
| --- | --- | --- | --- | --- | --- |
| 1. I know what health care is available ...   - - At the CSP   - In hospital   2. I think that the care provided at ... can really improve my child's health   - - At the CSP   - In hospital   3. I think that the care provided ... can indeed improve my child's health, even if he or she has a serious illness.   - - At the CSP   - In hospital   4. I think that the services available at the hospital are better able to meet my health needs and those of my child than those at the CSP.  Why? (Keywords) ……...................................................  5. If I want to take my child for treatment, I can make the decision to take him/her alone.  If not, who do you need to ask for permission? and why? .........................................................................  6. I think that the services provided are the same for everyone and that there is no discrimination...   - At the CSP - In hospital   7. There are cultural aspects that may prevent me from going...   - At the CSP - In hospital   8. The way health activities are organised and carried out is acceptable to me...   - At the CSP - In hospital   9. Your good relationship with the health workers ... makes you want to go there when you need to.   - - At the CSP   - In hospital   10. It is easy to get to me ... from my home   - At the CSP - In hospital   11. The opening hours and availability of people are sufficient to allow me to access health services when I need them...   - At the CSP - In hospital   12. The waiting time for my child to be picked up is usually long...   - At the CSP - In hospital   13. During my visits, I find that in general the medicines I need are readily available...   - At the CSP - In hospital   14. It costs me less to care for my child in hospital than it does to care for my child in the CSP.  15. Going to the SPC takes less time and has less impact on the daily activities I have to do than going to the hospital.  16. I have the financial means to have my child treated...   - At the CSP - In hospital     17. I am still able to pay for non-medical indirect costs (transport, accommodation, food, etc.) for care...   - At the CSP - In hospital   18. The care you can receive at ... is of good quality.   - At the CSP - In hospital   19. I am generally satisfied with the care my child receives when I go for a consultation...   - At the CSP - In hospital   20. I can communicate easily with health workers in the workplace.   - At the CSP - In hospital   21. When you take your child for treatment at ....., you generally understand the recommendations you receive from health professionals and are able to apply them   - At the CSP - In hospital | *□*  *□*  *□*  *□*  *□*  *□*  *□*  *□*  *□*  *□*  *□*  *□*  *□*  *□*  *□*  *□*  *□*  *□*  *□*  *□*  *□*  *□*  *□*  *□*  *□*  *□*  *□*  *□*  *□*  *□*  *□*  *□*  *□*  *□*  *□*  *□*  *□*  *□* | *□*  *□*  *□*  *□*  *□*  *□*  *□*  *□*  *□*  *□*  *□*  *□*  *□*  *□*  *□*  *□*  *□*  *□*  *□*  *□*  *□*  *□*  *□*  *□*  *□*  *□*  *□*  *□*  *□*  *□*  *□*  *□*  *□*  *□*  *□*  *□*  *□*  *□* | *□*  *□*  *□*  *□*  *□*  *□*  *□*  *□*  *□*  *□*  *□*  *□*  *□*  *□*  *□*  *□*  *□*  *□*  *□*  *□*  *□*  *□*  *□*  *□*  *□*  *□*  *□*  *□*  *□*  *□*  *□*  *□*  *□*  *□*  *□*  *□*  *□*  *□* | *□*  *□*  *□*  *□*  *□*  *□*  *□*  *□*  *□*  *□*  *□*  *□*  *□*  *□*  *□*  *□*  *□*  *□*  *□*  *□*  *□*  *□*  *□*  *□*  *□*  *□*  *□*  *□*  *□*  *□*  *□*  *□*  *□*  *□*  *□*  *□*  *□*  *□* | *□*  *□*  *□*  *□*  *□*  *□*  *□*  *□*  *□*  *□*  *□*  *□*  *□*  *□*  *□*  *□*  *□*  *□*  *□*  *□*  *□*  *□*  *□*  *□*  *□*  *□*  *□*  *□*  *□*  *□*  *□*  *□*  *□*  *□*  *□*  *□*  *□*  *□* |

***Free comments from the interviewer concerning the interview conducted. This can consist of transcribing something that struck him/her during the interview, a specificity, a remarkable element (e.g. an outstanding experience recounted by the accompanying person, a cultural barrier mentioned, an intriguing element, etc). It can also simply be a short overall assessment of the interview and the important facts mentioned.***

|  |
| --- |

# *Supplementary file 3: Time (in days) between first symptoms and arrival at the hospital according to visit or not to a PHC (A) and according to the recommendations of the healthcare system (B), ITINER'AIRE (N = 861).*


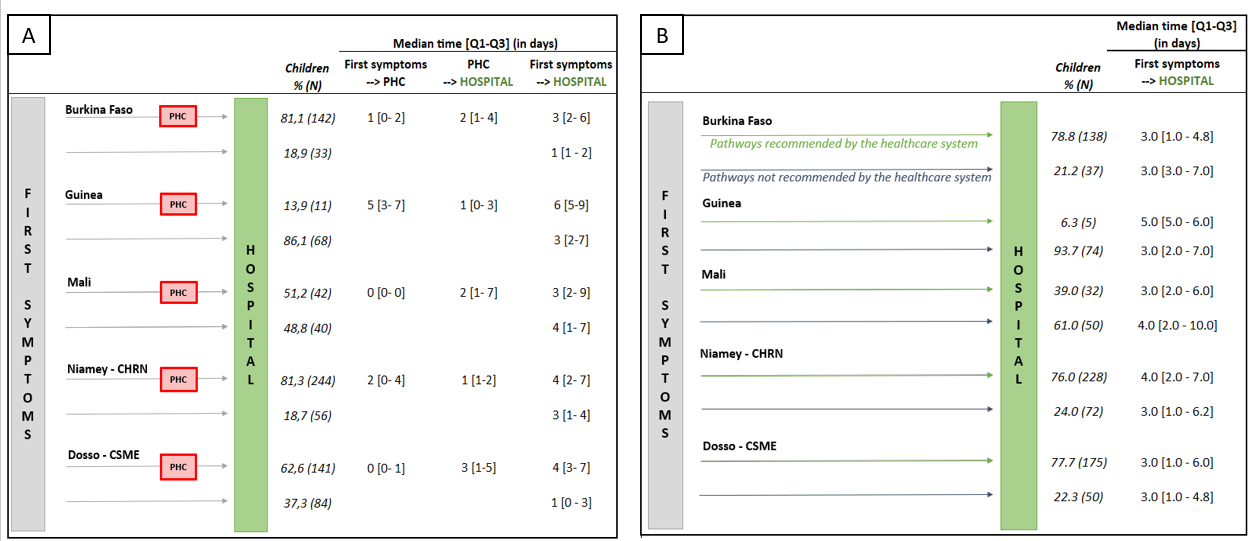


# *Supplementary File 4: Comparisons of the socio-demographic characteristics of patients according their attendance or not a PHC by countries.*

|  | **Burkina Faso (N=175)** | |  |
| --- | --- | --- | --- |
|  | **PHC visited**  **(N=142)** | **PHC not visited**  **(N=33)** | *p-value* |
| **Child’s gender, male N (%)** | 88 (62.0) | 22 (66.7) | 0.600 |
| **Child’s age, N (%)** |  |  | <0.001 |
| Less than 2 months | 39 (27.5) | 22 (66.7) |  |
| 2 - 59 months | 103 (72.5) | 11 (33.3) |  |
| **Child's caregivers, N (%)** |  |  | 0.025 |
| Mother | 95 (66.9) | 24 (72.7) |  |
| Father | 33 (23.2) | 2 (6.1) |  |
| Other family members | 14 (9.9) | 7 (21.2) |  |
| **Level of education of caregiver, N (%)** |  |  | 0.300 |
| None | 94 (66.2) | 22 (66.7) |  |
| Primary school | 22 (15.5) | 2 (6.1) |  |
| Secondary school or university | 26 (18.3) | 9 (27.3) |  |

|  | **Guinea (N=79)** | |  |
| --- | --- | --- | --- |
|  | **PHC visited**  **(N=11)** | **PHC not visited**  **(N=68)** | *p-value* |
| **Child’s gender, male N (%)** | 8 (72.7) | 48 (70.6) | 0.900 |
| **Child’s age, N (%)** |  |  | 0.700 |
| Less than 2 months | 2 (18.2) | 10 (14.7) |  |
| 2 - 59 months | 9 (81.8) | 58 (85.3) |  |
| **Child's caregivers, N (%)** |  |  | 0.400 |
| Mother | 6 (54.5) | 43 (63.2) |  |
| Father | 5 (45.5) | 18 (26.5) |  |
| Other family members | 0 (0.0) | 7 (10.3) |  |
| **Level of education of caregiver, N (%)** |  |  | 0.900 |
| None | 6 (54.5) | 33 (48.5) |  |
| Primary school | 1 (9.1) | 5 (7.4) |  |
| Secondary school or university | 4 (36.4) | 30 (44.1) |  |

|  | **Mali (N=82)** | |  |
| --- | --- | --- | --- |
|  | **PHC visited**  **(N=42)** | **PHC not visited**  **(N=40)** | *p-value* |
| **Child’s gender, male N (%)** | 25 (59.5) | 23 (57.5) | 0.900 |
| **Child’s age, N (%)** |  |  | 0.500 |
| Less than 2 months | 18 (42.9) | 14 (35.0) |  |
| 2 - 59 months | 24 (57.1) | 26 (65.0) |  |
| **Child's caregivers, N (%)** |  |  | 0.001 |
| Mother | 16 (38.1) | 25 (62.5) |  |
| Father | 13 (31.0) | 14 (35.0) |  |
| Other family members | 13 (31.0) | 1 (2.5) |  |
| **Level of education of caregiver, N (%)** |  |  | 0.031 |
| None | 29 (69.0) | 16 (40.0) |  |
| Primary school | 9 (21.4) | 16 (40.0) |  |
| Secondary school or university | 4 (9.5) | 8 (20.0) |  |

|  | **Niamey - Niger (N=300)** | |  |
| --- | --- | --- | --- |
|  | **PHC visited**  **(N=244)** | **PHC not visited**  **(N=56)** | *p-value* |
| **Child’s gender, male N (%)** | 149 (61.1) | 30 (53.6) | 0.300 |
| **Child’s age, N (%)** |  |  | 0.200 |
| Less than 2 months | 21 (8.6) | 8 (14.3) |  |
| 2 - 59 months | 223 (91.4) | 48 (85.7) |  |
| **Child's caregivers, N (%)** |  |  | 0.200 |
| Mother | 224 (91.8) | 54 (96.4) |  |
| Father | 2 (0.8) | 1 (1.8) |  |
| Other family members | 18 (7.4) | 1 (1.8) |  |
| **Level of education of caregiver, N (%)** |  |  | 0.002 |
| None | 157 (64.3) | 24 (42.9) |  |
| Primary school | 39 (16.0) | 9 (16.1) |  |
| Secondary school or university | 48 (19.7) | 23 (41.1) |  |

|  | **Dosso - Niger (N=225)** | |  |
| --- | --- | --- | --- |
|  | **PHC visited**  **(N=141)** | **PHC not visited**  **(N=84)** | *p-value* |
| **Child’s gender, male N (%)** | 70 (49.6) | 48 (57.1) | 0.300 |
| **Child’s age, N (%)** |  |  | <0.001 |
| Less than 2 months | 22 (15.6) | 52 (61.9) |  |
| 2 - 59 months | 119 (84.4) | 32 (38.1) |  |
| **Child's caregivers, N (%)** |  |  | <0.001 |
| Mother | 114 (80.9) | 44 (52.4) |  |
| Father | 2 (1.4) | 0 (0.0) |  |
| Other family members | 25 (17.7) | 40 (47.6) |  |
| **Level of education of caregiver, N (%)** |  |  | 0.700 |
| None | 86 (61.0) | 48 (57.1) |  |
| Primary school | 18 (12.8) | 10 (11.9) |  |
| Secondary school or university | 37 (26.2) | 26 (31.0) |  |

# *Supplementary file 5: Structures visited by children according to whether the illness is perceived by parents as simple or severe, ITINER'AIRE Study 2022 (N = 861)*

|  | **Burkina Faso (N=175)** | | | **Guinea (N=79)** | | | **Mali (N=82)** | | | **Niger - Niamey (N=300)** | | | **Niger - Dosso (N=225)** | | |
| --- | --- | --- | --- | --- | --- | --- | --- | --- | --- | --- | --- | --- | --- | --- | --- |
|  | Simple illness | Severe illness | *p* | Simple illness | Severe illness | *p* | Simple illness | Severe illness | *p* | Simple illness | Severe illness | *p* | Simple illness | Severe illness | *p* |
| PHC | 91% | 64% | *** | 84% | 54% | *** | 68% | 66% | NS | 89% | 87% | *NS* | 96% | 32% | ***** |
| Hospital | 5% | 96% | *** | 84% | 100% | *** | 21% | 95% | *** | 17% | 74% | ***** | 3% | 99% | ***** |
| TMP | 12% | 8% | * | 0% | 0% | NS | 29% | 10% | *** | 0% | 0% | *NS* | 0% | 0% | *NS* |
| Community health worker | 1% | 1% | NS | 1% | 1% | NS | 23% | 6% | *** | 0% | 0% | *NS* | 0% | 0% | *NS* |
| Private health centre | 1% | 2% | NS | 0% | 0% | NS | 7% | 9% | NS | 2% | 2% | *NS* | 0% | 0% | *NS* |
| Pharmacist | 3% | 0% | NS | 0% | 0% | NS | 9% | 2% | NS | 4% | 0% | **** | 0% | 0% | *NS* |
| Health post | 2% | 2% | NS | 9% | 5% | NS | 0% | 0% | NS | 0% | 0% | *NS* | 12% | 6% | ***** |

*PHC : Primary health centre, TMP : Traditional medicine practitioner*

*p (p-value) : Estimation of p-values using the Mac Nemar test, * p-value < 0,05 ** p-value < 0,01 *** p-value < 0,001 NS : Not significant*
